# Selective DNA-binding of SP120 (rat ortholog of human hnRNP U) is mediated by arginine-glycine rich domain and modulated by RNA

**DOI:** 10.1371/journal.pone.0289599

**Published:** 2023-08-04

**Authors:** Mary Miyaji, Shinji Kawano, Ryohei Furuta, Emi Murakami, Shogo Ikeda, Kimiko M. Tsutsui, Ken Tsutsui

**Affiliations:** 1 Department of Neurogenomics, Graduate School of Medicine, Dentistry and Pharmaceutical Sciences, Okayama University, Okayama, Japan; 2 Faculty of Science, Department of Biochemistry, Okayama University of Science, Okayama, Japan; Gandhi Insititute of Technology and Management, INDIA

## Abstract

A human protein heterogeneous ribonucleoprotein U (hnRNP U) also known as Scaffold attachment factor A (SAF-A) and its orthologous rat protein SP120 are abundant and multifunctional nuclear protein that directly binds to both DNA and RNA. The C-terminal region of hnRNP U enriched with arginine and glycine is essential for the interaction with RNA and the N-terminal region of SAF-A termed SAP domain has been ascribed to the DNA binding. We have reported that rat hnRNP U specifically and cooperatively binds to AT-rich DNA called nuclear scaffold/matrix-associated region (S/MAR) although its detailed mechanism remained unclear. In the present study analysis of hnRNP U deletion mutants revealed for the first time that a C-terminal domain enriched with Arg-Gly (defined here as ‘RG domain’) is predominantly important for the S/MAR-selective DNA binding activities. RG domain alone directly bound to S/MAR and coexistence with the SAP domain exerted a synergistic effect. The binding was inhibited by netropsin, a minor groove binder with preference to AT pairs that are enriched in S/MAR, suggesting that RG domain interacts with minor groove of S/MAR DNA. Interestingly, excess amounts of RNA attenuated the RG domain-dependent S/MAR-binding of hnRNP U. Taken together, hnRNP U may be the key element for the RNA-regulated recognition of S/MAR DNA and thus contributing to the dynamic structural changes of chromatin compartments.

## Introduction

hnRNP U is a highly abundant nuclear protein that binds to both DNA and RNA. This protein was originally isolated from HeLa cells as one of the components of hnRNPs and shown to be associated with poly(A)^+^ RNA [1]. On the other hand, a human nuclear protein termed scaffold attachment factor A (SAF-A) was discovered to bind specifically to scaffold/matrix attachment region (S/MAR) DNA [2]. Sequence determination of SAF-A demonstrated that these are identical proteins [3]. S/MARs are generally AT-rich noncoding sequences that facilitate the formation of specific functional loci in the nucleus [4,5]. We independently identified a rat ortholog of SAF-A in the nuclear scaffold preparation that selectively binds to S/MAR DNA and named it SP120 (scaffold protein 120 kDa) [6,7]. We use the term “hnRNP U” instead of SP120 throughout the present report although all the experiments were done with rat SP120. “SAF-box”, a short stretch of 31 amino acids at the amino-terminal region of SAF-A, was demonstrated to be responsible for the selective binding to S/MAR [8]. They immobilized SAF-box peptide (amino acid 1–45 of SAF-A) onto Sepharose beads and showed that the S/MAR-specific DNA binding to this peptide is highly dependent on its surface density, which suggested involvement of some cooperative mechanism. In a broader perspective study, homology search with amino acid sequences revealed a conserved motif called “SAP” in vast varieties of DNA-binding proteins including SAF-A, in which the SAF-box coincides with SAP [9]. We present here some evidence that the SAP domain (SAF-box) is also involved in the S/MAR-selective binding of rat hnRNP U, but its C-terminal region appears more essential for the full-range affinity.

**Fig 1:**
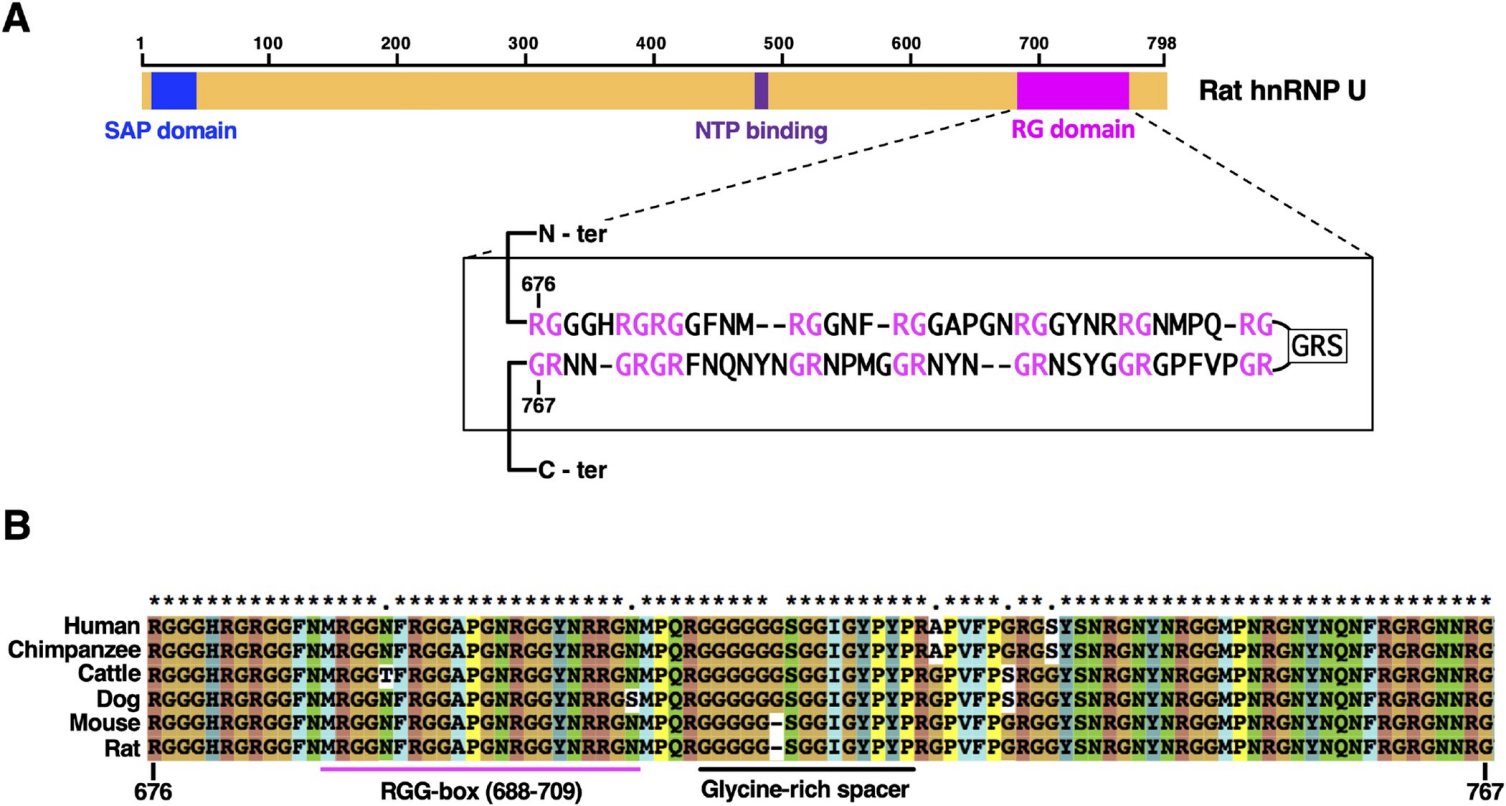
Domain structure of rat hnRNP U. (A) Domain diagram of rat hnRNP U used in this study. SAP (amino acids 8–42), NTP-binding (amino acids 478–485), and RG (amino acids 676–767) domains are indicated as colored boxes on full-length rat hnRNP U (amino acids 1–798). The RG domain is newly defined in this report whose amino acid sequence is shown in the box below. It contains a cluster of 16 RG dipeptides interrupted by the central glycine-rich spacer (GRS). The sequence is folded like a hairpin to highlight the symmetrical distribution of RGs. (B) Sequence alignment of the RG domain which is highly conserved in higher eukaryotes. RGG-box is originally described as a conserved motif in RNA binding proteins [10]. Glycine-rich spacer (GRS) is marked by a black line.

As a characteristic feature located in the C-terminal region of human hnRNP U, an RNA binding motif, termed RGG-box was specified within the C-terminal arginine and glycine-rich 112 amino acids [10]. We define here a wider region called ‘RG domain’ that is composed of symmetrically arranged 8x2 RGs flanking a glycine-rich spacer (Fig 1A). RG domain is highly conserved among mammals and harbors the RGG-box (Fig 1B). We have shown previously that a region slightly wider than the RG domain of hnRNP U is required for RNA-mediated interaction with DNA topoisomerase IIβ [11]. In general, RGG/RG motifs are wide-spread in more than 1,000 human proteins. As many proteins with RGG/RG motifs are RNA-binding proteins, this motif is considered to be involved in RNA-binding [12], although it is also reported to be involved in binding with double stranded DNAs [13].

Structurally RG domain is an intrinsically disordered region (IDR) [14], and can cause IDR-mediated phase separation [15]. Phase separation has been shown to be closely involved in cellular organization and regulation of nuclear structures [16,17]. It was suggested that microphase separation in the nuclear microenvironment can be switched between transcriptionally active and inactive states, that are reflected by ‘A’ and ‘B’ compartments observed in HiC experiments [18]. In the active microphase, sequence-specific multivalent DNA binding proteins are engaged in binding equilibrium with DNA to support transcription. Interestingly, several reports suggest that hnRNP U is required for maintaining the active (A) compartments [19,20]. In this context, RNA molecules and RNA-binding proteins may be main players in maintaining active compartments.

In this study, we constructed several deletion mutant proteins of hnRNP U and examined their DNA-binding and S/MAR-selective binding activities in a S/MAR vs non-S/MAR competitive binding scheme. Interestingly, deletion of RG domain disrupted the S/MAR selective DNA-binding of hnRNP U in soluble conditions. We also found a synergism between SAP and RG domains for the S/MAR-selective binding, which is interfered by excess amounts of RNA. Relationship between SAP and RG domains in the recognition of S/MAR by hnRNP U and a regulatory involvement of RNA will be discussed in view of the structural dynamism of nuclear matrix [20].

## Materials and methods

### Plasmid constructions

PCR primers for constructing plasmids used in this study are listed in S1 Table. His-Myc constructs of hnRNP U ΔSAP, ΔRG, NC, and NCΔRG were amplified with Phusion Hot Start DNA polymerase (NEB) by using pCold II-Myc-hnRNP U plasmid and its derivatives as templates [7]. The PCR products were cloned into pCold II-Myc vectors in–frame by swapping the full-length hnRNP U region of pCold II-Myc-hnRNP U. To amplify the DNA fragment encoding ΔRG and NC, PCR was performed in two steps. For the first step, DNA fragments encoding the N-terminal and the C-terminal parts of these mutants were amplified and the products were gel-purified. For the second step, PCR was performed using the primers in S1 Table and the mixtures of purified N-terminal and the C-terminal fragments prepared by the first step as templates. To amplify the DNA fragment encoding NCΔRG, PCR was performed in the same way as amplifying the DNA fragment encoding ΔRG by using pColdII-Myc-NC as a template. The DNA fragments encoding His-Myc-NC or His-Myc-NCΔRG were amplified using the primers listed in S1 Table with pColdII-Myc-NC or pColdII-Myc-NCΔRG as templates then re-cloned into pST39 vector [21]. pGEX3X-RG and pGEX3X-SAP, and pGEX3X-SAP-RG were prepared by cloning the PCR-amplified products in-frame into pGEX3X vector. The R to K substitution mutant, pCold II-Myc-RK, was constructed by PCR in three steps. For the first step, N-terminal and C-terminal parts of DNA fragments were amplified using primers and pColdII-Myc-hnRNP U as a template. Middle part containing RG domain was amplified from an artificial gene (supplied by Life Technologies) in which all arginine residues in RG domain were changed to lysine residues. For the second step, PCR was performed using primers and the mixture of purified 2 fragments, N-terminal, middle as templates, and then an amplified fragment (the N- and middle parts were fused) was gel purified. For the third step, PCR was performed using primers and the mixture of the fused fragment and C-terminal parts as templates.

To prepare S/MAR DNA and vector fragments for EMSA, plasmid DNAs were digested with restriction endonucleases under the conditions recommended by the supplier (New England Biolabs) and purified by phenol/chloroform extraction and ethanol precipitation and dissolved in TE buffer (10 mM Tris-HCl: pH8.0, 0.1 mM EDTA). The DNA fragments (pBS/*FTZ*) were generated by *Eco* RI digestion of pFTZ1[7]. The plasmid, pBS-*Kcnd2* intergenic region, was constructed as follows. *Kcnd2* gene intergenic region was amplified on rat genomic DNA with PCR primers shown in S1 Table. Rat genomic DNA was prepared from purified brain nuclei of two-week-old Wistar rats as described [22]. The experiment was conducted in accordance with protocols approved by the Animal Care and Use Committee of the Okayama University Medical School to minimize suffering in the animals. Rats were euthanized by exsanguination under anesthetic (intraperitoneal injection of pentobarbital sodium). The product DNA was purified and phosphorylated by T4 polynucleotide kinase (NEB) according to the manufacture’s protocol and cloned into *Eco* RV site of pBlueScript II (SK+). The DNA fragments used for EMSA assay (S1 Fig) were generated by *Bam* HI and *Xho* I digestions of pBS-*Kcnd2* intergenic region. A topoisomerase IIβ target #31 cloned in pZErO vector [7] was used to generate pZErO/*Sat* I DNA. The plasmid was digested with *Bam* HI and *Xho* I to separate vector and insert. A randomly cloned fragment of *E*. *coli* DNA (pBS-*E*. *coli* DNA) was digested with *Eco* RI and *Xho* I to generate pBS/*E*. *coli* DNA.

### Recombinant proteins

Preparations of His-Myc tagged recombinant proteins were performed as previously described [7], except that the lysates were incubated with RNase A (10 μg/ml, QIAGEN) and DNase I (40 u/ml, Takara) on ice for 30 min and the wash buffer containing 1 M NaCl instead of 0.5 M NaCl was used. To express His-Myc-NC and NCΔRG, *E*. *coli* cells BL21(DE3) were transformed with pST39-His-Myc-NC and pST39-His-Myc-NCΔRG and the transformed cells were grown in LB medium supplemented with 100 μg/ml ampicillin at 37°C. When the culture reached OD 0.3 (600 nm), 1mM IPTG was added and continued to cultivate at 37°C for 3 hours. Protein purification was conducted as described [7]. GST-fusion proteins were expressed in *E*. *coli* (Rosetta Blue (DE3) (Novagen)) and *E*. *coli* cells were recovered by centrifugation. The pellet was resuspended in an appropriate volume of lysis buffer (10 mM Tris-HCl (pH8.0), 500 mM NaCl, 0.5 mM PMSF, 1 mg/ml lysozyme) and incubate on ice for 15 min. Cells were disrupted by sonication (TOMY UD-201 with microtip). RNase A and DNase I were added to the lysate at a final concentration of 10 μg/ml and 40 u/ml respectively and incubated on ice for 30 min. Triton X-100 and DTT were added at final concentrations of 2% and 5 mM, respectively. The cleared lysates were obtained by centrifugation and stored into several aliquots at -80˚C. Protein amounts on beads were estimated by calibration to BSA standards on SDS-PAGE-stained gels. Raw images are shown in S4 Fig.

### EMSA

Recombinant hnRNP U-WT and its mutants were incubated at 37°C for 30 min with 25 ng of equimolar mixture of vector and insert DNA fragments in 15 μl of reaction mixture containing 35 mM Tris–HCl (pH 8.0), 72 mM KCl, 5 mM MgCl_2_, 5 mM dithiothreitol (DTT), 5 mM spermidine, and 50 μg/ml BSA. Samples were then subjected to electrophoresis on a 1% SeaKem GTG agarose gel containing 10 mM MgCl_2_ in 0.5× TBE (Tris–borate–EDTA) buffer. The tank buffer (0.5× TBE) did not contain MgCl_2_. After staining with 0.5 μg/ml EtBr, DNA bands were recorded on VersaDoc^TM^ (Bio-rad) and quantified by densitometry. Uncropped original agarose gel images are also shown in S5 Fig.

### On-bead binding assay

Bead-bound GST-fusion proteins were prepared by incubating aliquots of glutathione-Sepharose beads with the lysate containing varying amounts of GST-fusion proteins at 25˚C for 1 h. Beads were washed 3 times with wash buffer (10 mM Tris-HCl (pH 8.0), 500 mM NaCl, 0.1% Triton X-100) and then three times with binding buffer (50 mM Tris-HCl (pH8.0), 120 mM KCl, 10 mM MgCl_2_, 30 μg/ml BSA, 0.5 mM EDTA, 0.5 mM DTT, 0.1% Triton X-100). DNA fragments (50 ng or 100 ng of pBS/*FTZ*) in binding buffer were added and incubated at 25˚C for 1hr with mixing. The unbound DNA was separated by centrifugation. Beads were washed 3 times with the binding buffer and deproteinized by proteinase K (Roche) treatment. The released DNA was separated by centrifugation and recovered as bound fraction. Unbound and bound DNAs were purified and analyzed by 1% agarose gel electrophoresis. To examine the inhibitory effects of netropsin, pBS/*FTZ* (100 ng) was preincubated with fixed amounts of netropsin in the binding buffer at 25˚C for 30 min. The preincubated DNA fragments were added to 10 μl of beads coupled with 16.5 pmol of GST-RG. The subsequent processes were performed as described above.

### Aggregation assay

DNA fragments (amounts of pBS/*FTZ* indicated in Figure legend) were incubated with purified His-Myc-hnRNP U proteins in 10 μl of aggregation buffer containing 50 mM Tris-HCl (pH 8.0), 120 mM KCl, 10 mM MgCl_2_, 0.5 mM EDTA, and 30 μg/ml BSA at 37˚C for 30 min. Samples were separated into pellet and supernatant by centrifugation at 20,000 xg for 10 min at 4˚C. Pellets were resuspended in 10 μl of aggregation buffer. Two μl of reaction mixture containing 4% SDS and 2 mg/ml Proteinase K (Roche) were added to the supernatants and the pellets and incubated at 55˚C for 30 min. In the reactions containing RNA, samples were treated with 10 μg/ml RNase A at 37°C for 10 min to degrade RNA before the addition of SDS and Proteinase K. The samples were subjected to 1.0% agarose gel. DNA bands were detected by staining with GelRed Nucleic Acid Gel Stain (Biotium) and quantified by band densitometry using Image J software. Total RNA was purified from HEK293 cells by using RNeasy Mini kit (Qiagen). The total RNA was fractionated into poly A (+/-) RNA using Dynabeads mRNA Purification Kit (Invitrogen, DYNAL). To investigate the effect of salt concentration on the complex formation, pBS/*FTZ* (25 ng) was incubated with His-Myc-hnRNP U proteins in 25 μl of buffer containing 10 mM Tris-HCl (pH8.0), 10 mM MgCl_2_, 1 mM DTT, 1% glycerol and varying NaCl concentrations (Fig 6A and 6B).

## Results and discussion

### Detection of S/MAR-selective binding activities of hnRNP U by mobility shift assay (EMSA)

To analyze the S/MAR-selective binding of hnRNP U, we employed magnesium-agarose electrophoretic mobility shift assay (EMSA) [23]. Plasmids harboring S/MARs or *E*. *coli* DNA were digested with restriction enzyme to liberate inserts from the vector portion, resulting in equimolar mixtures of inserts and vector DNA fragments. Vector DNA serves as a non-S/MAR internal control. After purification, the DNA mixture was incubated with purified recombinant hnRNP U protein and separated by agarose gel electrophoresis in the presence of 10 mM magnesium chloride, which is required for the selective binding of S/MAR with hnRNP U [6]. hnRNP U complexed with DNA forms large aggregates that fails to migrate into gel. Therefore, the amount of bound DNA was estimated from the decreased band densities when compared to protein-free DNA bands (leftmost lanes in S1A Fig).

*FTZ* is a classical S/MAR isolated from the upstream region of Drosophila *Fushitarazu* gene [24]. The *FTZ* insert disappeared completely in the presence of 40 nM hnRNP U whereas the vector band (V) persisted, indicating that the protein binds to *FTZ* with high selectivity (S1B Fig). Upstream intergenic region of *Kcnd2* gene and rat *Sat* I repeats were obtained previously from DNA clones for topoisomerase IIβ action sites and found to be AT-rich S/MARs that bind to hnRNP U [7]. These DNA fragments also showed selective binding to hnRNP U as compared to the vector DNA. The C_50_ value reflecting relative affinities of S/MARs toward hnRNP U was estimated from the densitometric profiles (S1B Fig). As shown under the gel images of S1A Fig, C_50_ values roughly increase along with the GC-content of insert DNA fragments. This is consistent with our previous finding that the S/MAR-selective binding of hnRNP U positively correlates with the coverage rate of short A/T-tracts [7]. It is possible that not just GC-content, but also local density of A/T-tracts affects the affinity. The reason why C_50_ values for the same vector fragments vary somewhat in different insert combinations is not clear. These results indicated that magnesium-agarose EMSA is a useful tool for evaluating the AT-rich S/MAR-selective binding of hnRNP U in soluble conditions.

### RG domain is required for proper S/MAR-selective binding of hnRNP U in soluble conditions

**Fig 2:**
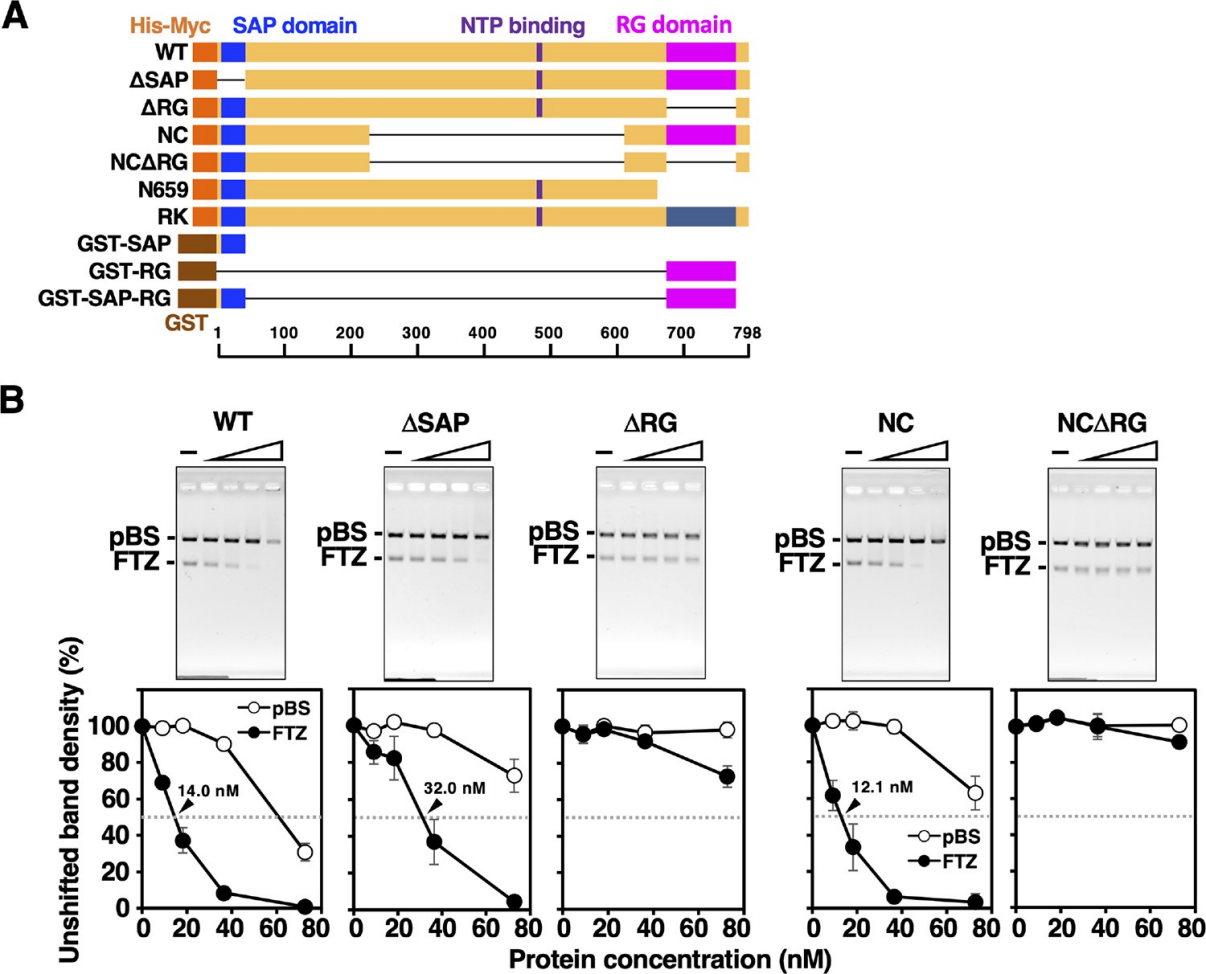
DNA-binding activities of domain deletion mutants. **(**A) Illustration of wild type (WT) and deletion mutants. The orange and brown boxes at the N-terminus represent His-Myc and GST tags, respectively. (B) Gel images from competitive magnesium-agarose EMSA are shown with band density plots. Concentrations for WT, ΔSAP, ΔRG, NC, and NCΔRG were varied. Error bars indicate mean ± standard deviation (SD) from three independent experiments. Half-maximal concentrations (C_50_) for WT, ΔSAP, and NC are indicated by arrowheads.

To determine the domain (s) of hnRNP U required for the S/MAR-selective binding, we constructed several deletion mutants (Fig 2A). SAP domain is a well-conserved region that specifically recognizes S/MAR DNA [8]. RG domain of the rat hnRNP U is a newly defined region (amino acid 676–767) that contains 16 Arg-Gly dipeptide sequences symmetrically positioned around the central glycine-rich spacer (Fig 1A). RG domain is highly conserved in mammals and contains the motif termed RGG-box that interacts with RNA [10] (Fig 1B). RGG or RG motifs exist in more than 1,000 human proteins and claimed to be involved in numerous physiological processes such as transcription, pre-mRNA splicing, DNA damage signaling, mRNA translation, and regulation of apoptosis [12]. We speculate that the whole structure of RG domain with regularly spaced RGs, not just repetitive short RGG/RG motifs, is important for the function of hnRNP U.

We compared the S/MAR-selective binding activities of hnRNP U deletion mutants using *FTZ* as a typical S/MAR (Fig 2B). A deletion mutant of SAP domain (ΔSAP in Fig 2A) had a slightly weaker but significant S/MAR-selective binding activity (C_50_: 32 nM) as compared to WT (C_50_: 14 nM). Although the SAP domain has been regarded as the region responsible for the S/MAR-selective binding of SAF-A by using immobilized proteins [8], these results indicate that other region(s) within ΔSAP is involved in the S/MAR-selective binding. To this end, we first examined a deletion mutant of RG domain (ΔRG). ΔRG showed a significantly decreased DNA-binding activity, being unable to determine C_50_ within the range of protein concentrations tested. Thus, RG domain is essential for the proper S/MAR-selective binding of hnRNP U in solution. To assess the role of the central region, we constructed the NC mutant in which the central region with putative ATPase domain (shown by ‘NTP binding’) was removed (Fig 2A). Remarkably, NC mutant had almost the same activity as WT, indicating that the central region is not required for S/MAR-selective binding of hnRNP U. On the other hand, NCΔRG showed a very low binding activity like ΔRG. Therefore, we reaffirmed that RG domain is essential for the proper S/MAR DNA binding of hnRNP U under soluble conditions.

### RG domain itself has selective and strong S/MAR-binding activity

The DNA-binding activity of the SAP domain is weak in solution but when immobilized on beads, it exhibited a high affinity toward S/MAR, depending positively on its surface density [8]. Our observation with ΔRG and NCΔRG mutants may suggest that the role of RG domain is simply increasing the local concentration of SAP domain to exert S/MAR-selective binding. Otherwise, it is also possible that the RG domain itself binds directly to S/MAR DNA under these conditions. Although RG domain can be regarded as an RNA-binding domain, gene ontology analysis showed that about half of the proteins with clustered RGG motifs are associated with both RNA- and DNA-binding functions [12].

**Fig 3:**
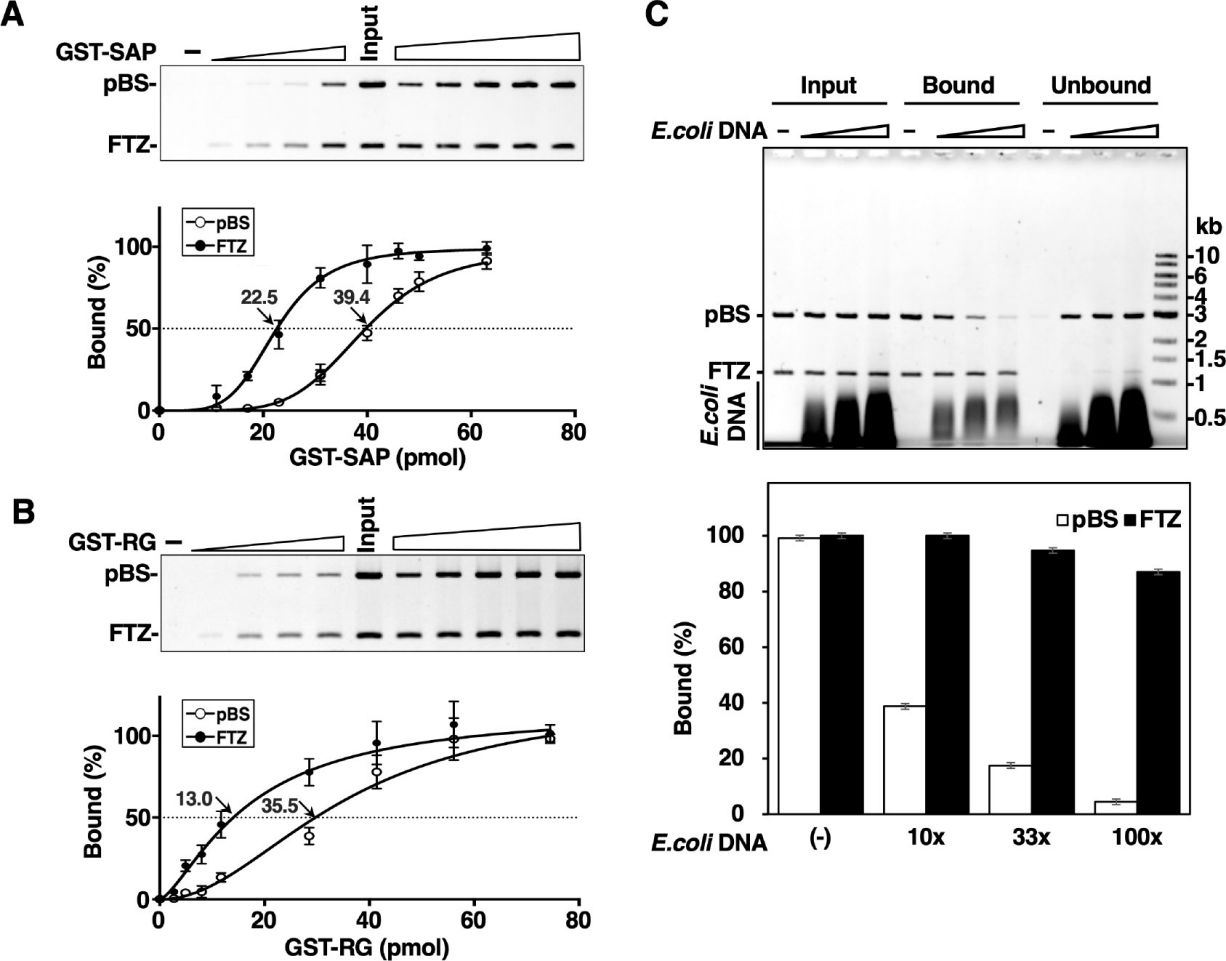
Comparison of DNA-binding activities of SAP and RG domains by on-bead DNA binding assay. (A) On-bead binding assay of SAP domain (amino acids 1–44). Fixed volume (2 μl) of glutathione-Sepharose beads coupled with increasing amounts of GST-SAP protein were incubated with 100 ng (37.3 fmol) of pBS/*FTZ*. Bead-bound DNA was analyzed by agarose gel electrophoresis. The quantified band densities relative to those of input are plotted against the amounts of GST-SAP on beads. Error bars indicate mean ± SD from at three independent experiments. In drawing the regression curve, a nonlinear curve fitting program (Graphpad prism) was applied to mean values. (B) On-bead binding assay of RG domain (amino acids 676–767). The same procedure as above was employed. Error bars indicate as described above. (C) On-bead binding assay of RG domain with or without excess amounts of *E*. *coli* DNA against 50 ng of pBS/*FTZ* (10, 33, and 100-folds). GST-RG (56 pmol) on 7.5 μl beads was used for these experiments. The GC content of pBS and *E*. *coli* DNA fragments used as competitor was 50.4% and 50.8%, respectively. Error bars indicate mean ± SD from three independent experiments.

To compare DNA-binding activities of RG and SAP domains directly, they were expressed as GST-fusion proteins and immobilized at varying concentrations on glutathione-Sepharose beads. Using this on-bead binding assay, we employed the competitive binding scheme with *FTZ* S/MAR plasmid as in Fig 2B (Fig 3). The immobilized SAP domain showed a selective binding toward S/MAR (Fig 3A), which is consistent with the previous report [8]. The immobilized RG domain also showed selective binding to S/MAR, indicating that this domain alone can discriminate S/MAR from non-S/MAR DNAs (Fig 3B). Although RG domain showed higher affinity to *FTZ* compared to SAP as judged from the lower half saturation dose (13.0 pmol vs. 22.5 pmol), overall selectivity for S/MAR appears to be lower than SAP, whose binding is cooperative in nature as reflected by the sigmoidal binding curve (Fig 3A). Interactions between SAP domains bound to neighboring sites on DNA may cause a strong selectivity by a mass binding mode [4]. The dissociation constant (K_d_) obtained from a nonlinear regression analysis on Fig 3A and 3B coincided with the C_50_, and the Hill coefficients were 4.6 and 1.8 for SAP and RG, respectively. This indicates that the binding affinity of GST-RG is higher than GST-SAP but with less cooperativity.

To further analyze the S/MAR-selective binding of RG domain, excess amounts of short *E*. *coli* DNA fragments (<1 kb, GC% = 50.8) were added as a competitor to the on-bead binding assay with pBS/*FTZ* (Fig 3C). A saturating dose of RG domain fixed on beads bound all input DNA fragments. As increasing the added competitor, binding of non-S/MAR DNA (pBS, GC% = 50.4) decreased whereas S/MAR (*FTZ*, GC% = 32.7) remained bound to GST-RG even in the presence of 100-fold excess amount of *E*. *coli* DNA against pBS/*FTZ* (Fig 3C). Taken together, RG domain itself has a strong DNA-binding activity with significant S/MAR-selectivity. This may be the reason why hnRNP U selectively binds to S/MARs under soluble conditions even if SAP domain was deleted (Fig 2B).

### RNA modulates the DNA selectivity of hnRNP U

**Fig 4:**
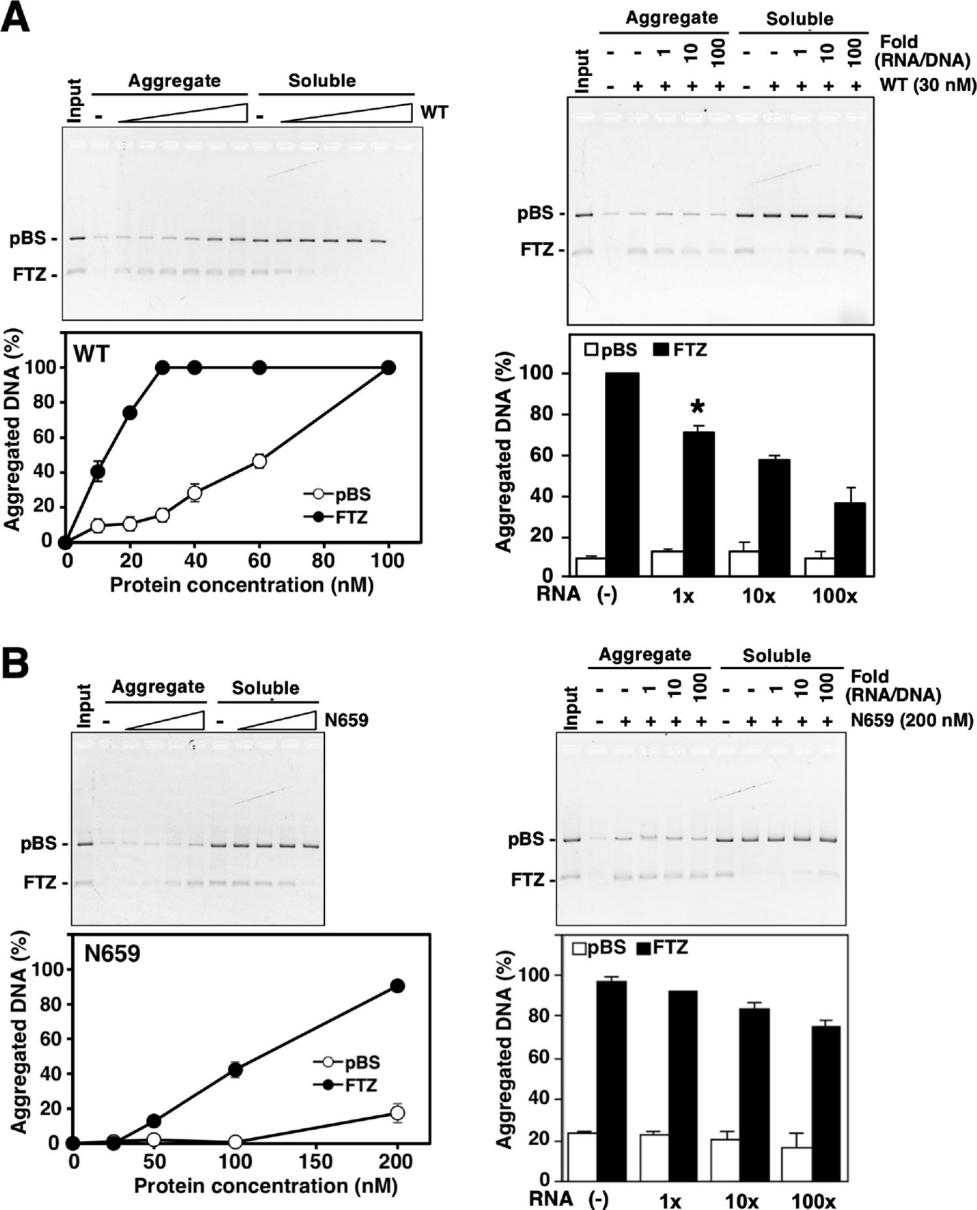
Effects of RNA on S/MAR-selective binding of hnRNP U. Aggregation assays using 20 ng of pBS/*FTZ* incubated with varying dose of recombinant hnRNP U proteins in the presence or absence of RNA. After the binding reaction, reaction mixture was centrifuged to separate into pellet (aggregate) and supernatant (soluble) fractions. Gel images and densitometric plots are shown in pairs. (A) Increasing concentrations of hnRNP U (WT) alone (left panels). The same experiment was done at 30 nM (right panels) of WT with or without excess amounts of total RNA (indicated by ‘fold’ against pBS/*FTZ* DNA). Bars plotted are average of 3 experiments with standard deviation. The decrease in aggregated DNA at 1x fold RNA was statistically significant by Student’s *t* test (marked by asterisk, *p*<0.01). (B) Increasing concentrations of N659 alone (left panels). The same experiment was done at 200 nM of N659 with or without excess amounts of total RNA (right panels). Error bars indicate mean ± SD from three independent experiments. No statistical significance in the reduction of aggregated DNA was detected at 1x RNA (*p*<0.05).

It is commonly accepted that the C-terminal region of hnRNP U containing RGG-box has an RNA-binding activity [10]. We next investigated whether RNA affects the DNA-binding and S/MAR-selectivity of hnRNP U. To examine the effect(s) of RNA on the hnRNP U-S/MAR interaction, we employed aggregation assays in which large complexes formed by hnRNP U and nucleic acids were separated by centrifugation into pellet and supernatant fractions, where the binding of hnRNP U to DNA but not RNA forms large pelletable aggregates [3]. Total cellular RNA purified from HEK293 cells was applied as an RNA source (Fig 4). We have shown previously that hnRNP U and topoisomerase IIβ forms a complex in the presence of total RNA, in which C-terminal region of hnRNP U was essential [11].

In the absence of hnRNP U, input DNA was recovered mainly in supernatant (Fig 4A, left panels). Amount of aggregated *FTZ* fragment increased sharply with increasing concentration of hnRNP U-WT. At a low concentration of hnRNP U-WT (30 nM), almost all *FTZ* but very little pBS fragments were aggregated into pellets, indicating the binding occurs in a S/MAR-selective manner. At a higher concentration of hnRNP U-WT (100 nM), however, almost all input DNA (both *FTZ* and pBS) were aggregated without any selectivity. In the presence of 30 nM WT (Fig 4A, right panels), the amount of aggregated *FTZ* fragment decreased with increasing amounts of RNA added, implying that the effect of RNA is competitive in nature since the binding sites for DNA and RNA overlap in the RG domain of hnRNP U.

To confirm that RG domain is responsible for the RNA effects, we prepared N659 that lacks the C-terminal region including RG domain (Fig 2A). N659 required much higher concentration to attain the same level of *FTZ* aggregation, almost ten times compared to WT (Fig 4B, left panels). This is consistent with the EMSA result with the deletion mutant lacking RG domain (Fig 2B). Therefore, the RNA effect had to be tested at a higher concentration of 200 nM (Fig 4B right panels). Aggregated *FTZ* by binding to N659 was only slightly reduced even at 100-fold excess amount of RNA. As the binding of WT to *FTZ* was significantly suppressed with the equal amount of RNA (Fig 4A, right panels), these results support the notion that RG domain is involved in the selective binding of S/MAR DNA, which is attenuated by the competitive binding of RNA on the same domain.

**Fig 5:**
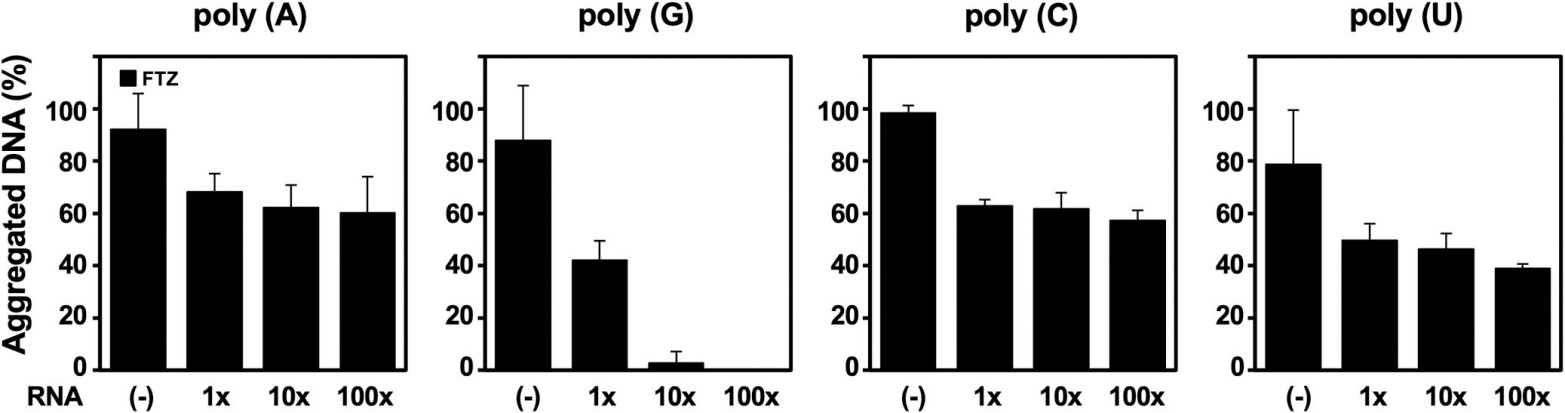
Effects of synthetic homoribopolymers on S/MAR-selective binding of hnRNP U. Aggregation assays using 20 ng of pBS/*FTZ* incubated with 20 nM of WT protein in the presence and absence of synthetic homoribopolymers that are indicated on top of the Figure. Added RNA amounts shown on bottom are expressed by ‘fold’ against pBS/*FTZ* DNA. Error bars indicate mean ± SD from three independent experiments.

We have shown previously that C-terminal domain (amino acid 612–798) of hnRNP U is associated with poly(A)^+^ RNA [11]. Random cloning and sequencing of the associated RNA revealed partial sequences of various mRNAs. Interestingly, poly(A)^+^ RNA was replaceable by synthetic homopolymer poly (G), but not by other homopolymers, poly (A/C/U). Recently RGG domain of hnRNP U was reported to bind specifically to G-quadruplex RNA [25]. Poly (G) sequences have a high propensity to form G-quadruplex structure [26], and a transcriptome-wide search detected frequent G-quadruplex structures in poly(A)^+^ RNA [27]. In fact, poly (G) most efficiently inhibited the S/MAR-binding of hnRNP U in the aggregation assay (Fig 5). Although effective RNA components *in vivo* remain to be determined, the competitive binding between S/MAR DNA and RNA with respect to the RG domain of hnRNP U may play regulatory roles in the nuclear environment through microphase separation. As both S/MAR DNA and RNA are composed of multiple molecular species, their enormous combination would substantiate subtle levels of regulation.

### Arginine residues in RG domain are important for proper S/MAR-binding activity of hnRNP U

**Fig 6:**
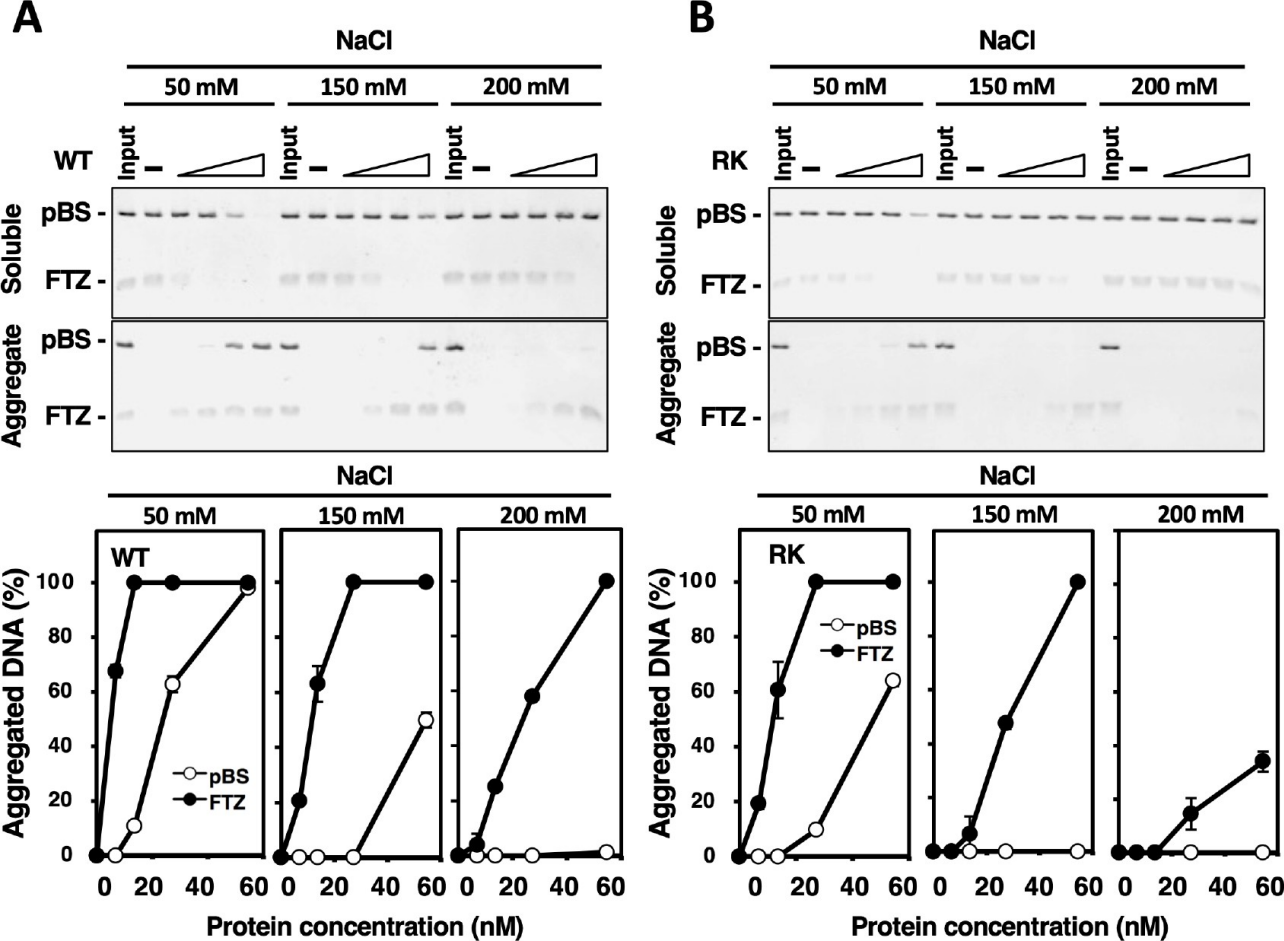
Effects of total substitution of arginines to lysines in the RG domain (RK mutant) on S/MAR-binding activity of hnRNP U. Aggregation assays with WT (A) and RK mutant (B) using 25 ng of pBS/*FTZ* at different NaCl concentrations (50, 150 and 200 mM). Gel images from aggregation assays are shown with band density plots below. Error bars indicate mean ± SD from three independent experiments.

Abundance and regular positioning of arginine residues in RG domain suggests that the guanidino group (or its positive charge) of arginine is responsible for the selective binding of hnRNP U to S/MAR DNAs. To test this, a mutant protein named RK was constructed, in which all arginines were substituted with lysines (a positive charge analogue of arginine), and the aggregation assay was performed at increasing salt concentrations to compare the stability of binding (Fig 6). At 50 mM NaCl, RK mutant also showed S/MAR-selectivity but its affinity toward *FTZ* was lower than WT as the protein concentration required to aggregate the same amount of DNA was doubled. RK mutant showed much lower affinity at higher NaCl concentrations (compare the aggregated DNA with 30 nM protein at 150 mM NaCl, for instance). These data may indicate that in RK mutant the interaction between DNA is mainly ionic through the e-amino group of lysine but in WT protein hydrogen bonding of the guanidino group of arginine between base pairs may enable the strong and specific interaction. Thus, in physiological ionic conditions, arginine residues in RG domain are likely to be important for stable interaction between hnRNP U and S/MAR DNA.

### Netropsin inhibits the S/MAR-selective binding of RG domain

S/MARs are generally AT-rich sequences enriched with short A/T-tracts that are recognized by S/MAR-binding proteins. This interaction is suppressed specifically by distamycin A, an antibiotic known to inhibit transcription [28]. Distamycin has a protonated nitrogen atom as in the guanidino group of arginine. To see whether similar mechanism is involved in the S/MAR-selective binding of hnRNP U, we used netropsin, a compound analogous to distamycin. Netropsin was shown to be a minor groove binder with AT pair preference [29]. It was also reported that narrow minor grooves of DNA are often associated with A/T-tracts and thus expressing enhanced negative electrostatic potentials, which stabilize the insertion of arginine residues in varieties of protein–DNA complexes [30].

**Fig 7:**
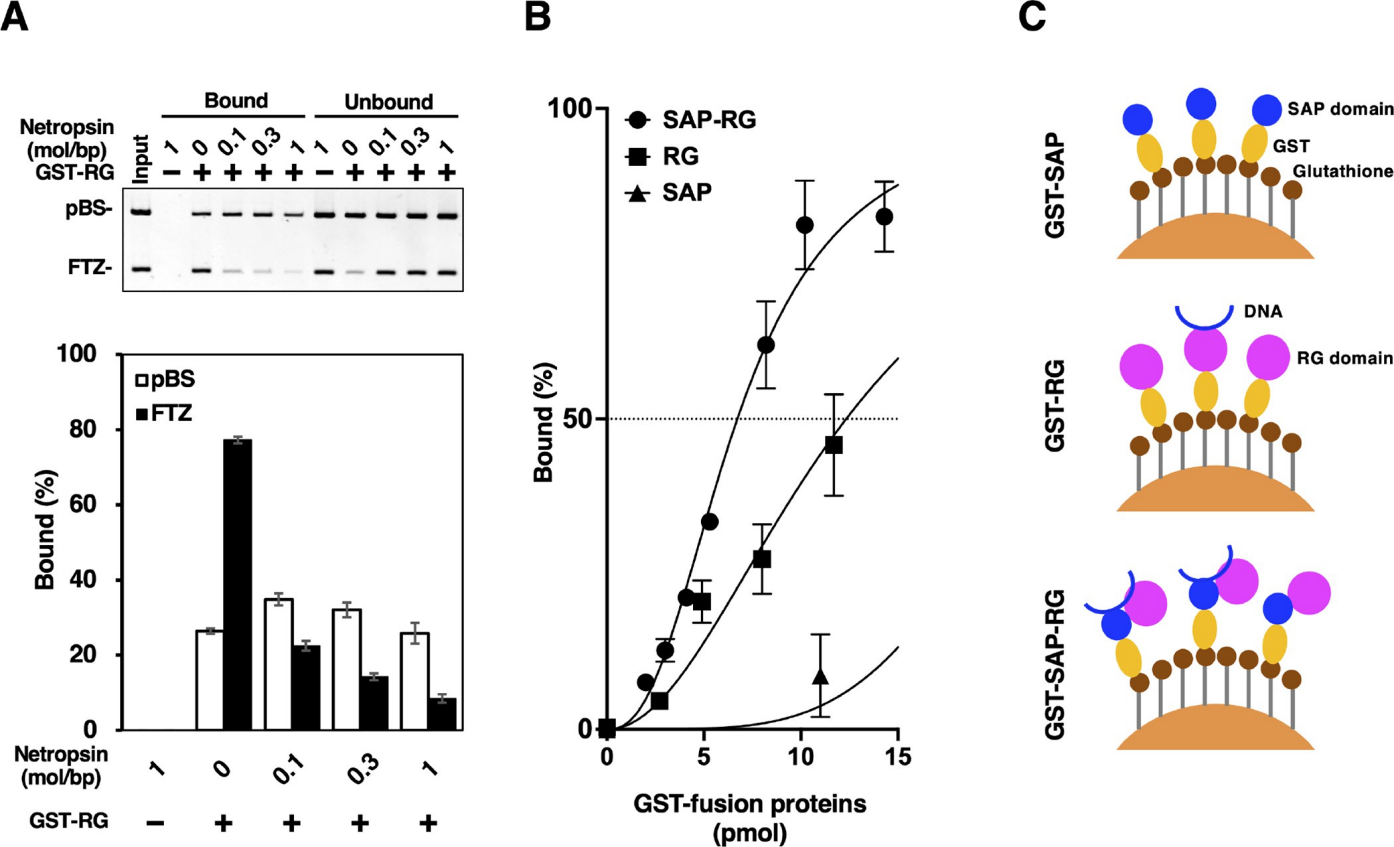
Effects of netropsin on the binding of *FTZ* to RG domain and possible synergism between SAP and RG domains. (A) Effects of netropsin on the selective binding of *FTZ*. For on-bead binding assays, GST-fusion proteins immobilized on glutathione-Sepharose beads (7.5 μl) was incubated with 100 ng of pBS/*FTZ* (37.3 fmol) and the bead-bound DNA fragments were analyzed. pBS/*FTZ* was pre-incubated with or without netropsin at the molar ratio indicated in the Figure (drug vs base pair in DNA). GST-RG (15 pmol) was used for the assays. Error bars indicate mean ± SD from three independent experiments. (B) On-bead binding assay of GST-SAP-RG. The same procedure as Fig 3A and 3B was employed. Data points for GST-SAP and GST-RG are transcribed from Fig 3A and 3B, respectively, and overlayed on the same Figure. Error bars indicate mean ± SD from three independent experiments. In drawing the regression curve, a nonlinear curve fitting program (Graphpad prism) was applied to mean values. (C) Schematic representation of the mechanism for synergistic binding.

It is likely, therefore, that arginine residues within RG domain interact with minor grooves of S/MAR DNA at the A/T-tracts. If so, it is expected that netropsin would antagonize the S/MAR-selective binding of RG domain. The on-bead binding assay demonstrated that the S/MAR-binding to RG domain (15 pmol) was selectively inhibited in a dose-dependent manner by pretreatment of DNA with netropsin (Fig 7A). The result supports the notion that RG domain of hnRNP U recognizes the minor groove of S/MAR through its arginine residues.

### A synergistic action between RG and SAP domains

Since DNA-binding activities of SAP and RG domains were assessed separately (Fig 3A and 3B), we next investigated the activity when these two domains coexist on the same molecule (GST-SAP-RG) to see whether synergistic effects are observed under the same conditions (Fig 7B). Regression analysis showed that K_d_ and Hill coefficient for GST-SAP-RG are 6.7 and 2.5, respectively. If SAP and RG domains bind DNA independently in GST-SAP-RG, the *FTZ* DNA binding will be additive. However, the result indicated that the level of bound DNA far more exceeds the sum of bound DNA with these domains alone and, more importantly, with higher binding affinity as reflected by the smaller K_d_ value, which is 13.0 and 22.5 for GST-RG and GST-SAP, respectively. This is a good indication that a synergistic interaction between SAP and RG domains is operating in the same molecule (shown schematically in Fig 7C). These domains may occupy close positions intramolecularly in native hnRNP U molecule although their direct physical association remains to be shown. A 3D structure of rat hnRNP U predicted by AlphaFold, a highly accurate protein structure prediction tool [31], showed that SAP forms a compact domain with two a-helixes, but RG domain takes a disordered and extended structure (S3 Fig). These domains indeed can come close together because of the presence of a long stretch of IDR between SAP and central globular domains.

**Fig 8:**
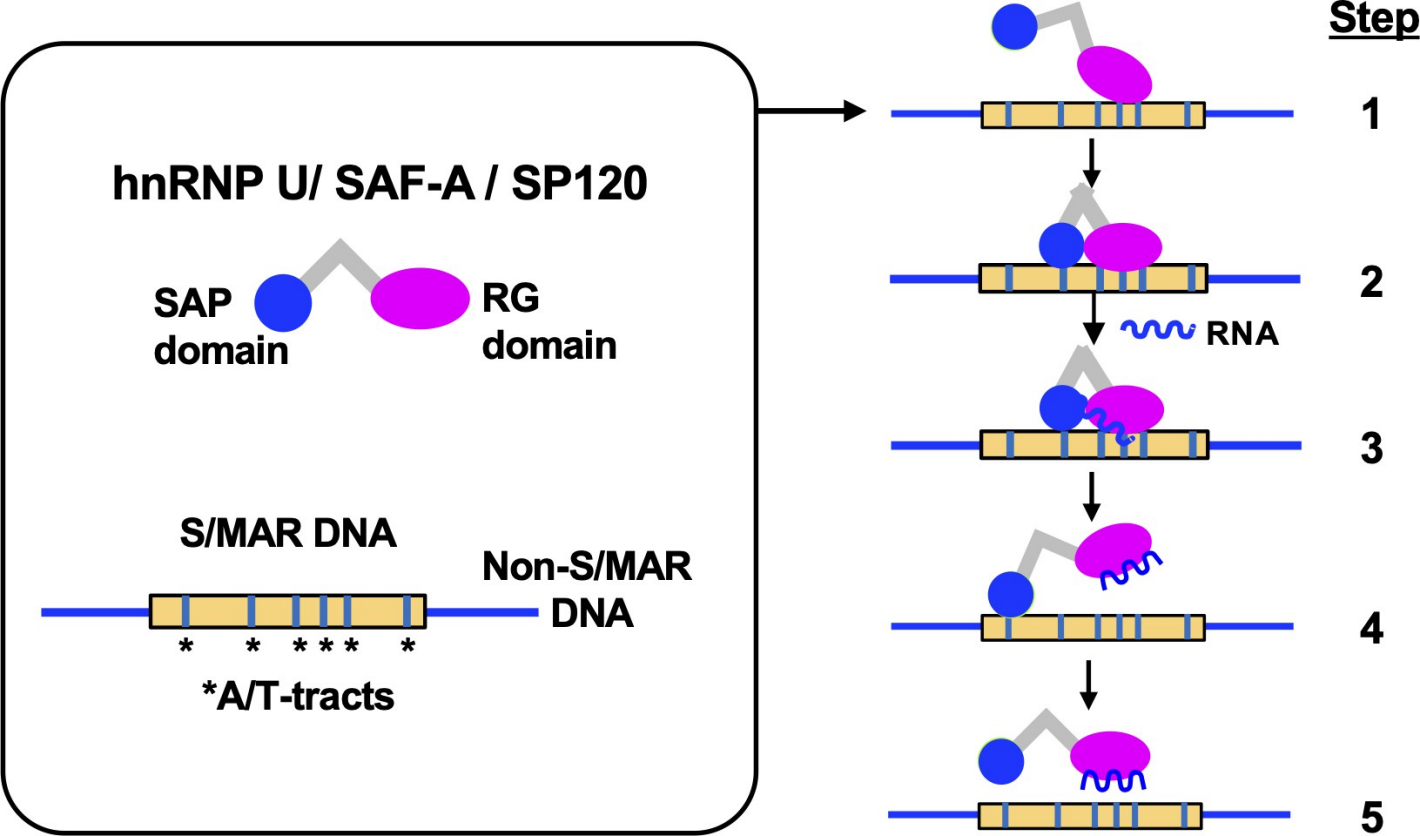
Schematic representation of the binding mechanism between hnRNP U and S/MAR DNA. To aid understanding, the binding process is expressed stepwise, which may not be the case in actual environment. Step1: RG domain recognizes and binds to A/T-tracts of S/MAR DNA. Step2: SAP domain interacts with RG domain and secure the binding. Step3: Ambient RNA binds to RG domain. Step4: RG domain is released from DNA and the interaction between SAP domain is disrupted. Step5: SAP domain with limited affinity is dissociated from DNA and hnRNP U entire molecule is released.

It is important to discriminate these domains with respect to the mode of interaction with S/MAR DNA. SAP domain requires multiple copies to form a stable association with single molecule of S/MAR (thus autosynergistic), but RG domain may require much less copies. The difference should be related to their domain size and amino acid composition. RG domain is about twice the size of SAP domain and is much enriched with basic amino acids, especially arginine. Arginine residues are supposed to interact directly with short A/T-tracts that are abundant in S/MAR DNA [7]. In contrast, molecular mechanism for the S/MAR-selectivity of SAP domain has never been proposed at this level. We speculate that in native hnRNP U molecule RG domain is the primary determinant of S/MAR recognition and intramolecular SAP domain ensures the binding. A plausible binding mechanism involved here is shown graphically in Fig 8.

## Conclusions and perspective

In this study, we demonstrated unequivocally for the first time that the RG domain of hnRNP U possesses S/MAR-selective DNA binding activity by itself, which had been attributed solely to the SAP domain. Previously known functions of hnRNP U RG domain are localization of hnRNP U to the nuclear matrix [32], interaction with topoisomerase IIβ [11], and association with inactive X chromosome [33,34], for which all RNA binding is considered important. The present study thus adds one more function to RNA, that is the modulation of S/MAR-selective DNA binding of hnRNP U.

A significant proportion of noncoding RNAs are highly repetitive RNAs like in LINE-1 transcripts and are associated with nuclear scaffold/matrix in euchromatin regions [35]. They presented evidence for involvement of SAF-A in the retention of RNA. Recent report showed that SAF-A interacts with chromatin-associated RNAs via its “RGG domain” (amino acid #695–797) to regulate interphase chromatin structures [20]. ATP binding/hydrolysis by the ATPase domain (designated ‘NTP binding’ in Fig 1A) and RNA-binding state of “RGG domain” were thought to be involved in oligomerization of SAF-A and the interconversion of a large-scale chromatin folding that affects the transcription of neighboring genes. More recently, it is proposed that a phenomenon called ‘phase separation’ is operating in the formation of subnuclear compartments like nuclear bodies and nucleoli, where RNA is one of the major role-players. In view of this emerging concept of nuclear organization, the multifunctional protein hnRNP U/SAF-A/SP120 can now be regarded as a leading player of dynamic nuclear transactions [20]. Our finding that RNA attenuates the interaction between hnRNP U and S/MAR DNA should be important in its regulatory function.

## Supporting information

S1 Fig S/MAR-selective binding activity of hnRNP U detected by magnesium-agarose EMSA. **(**A) Competitive EMSA analysis with equimolar mixtures of vectors and featured inserts. Twenty-five ng each of DNA were cleaved into vector (V) and insert (I) fragments, incubated with varying amounts of tag-purified hnRNP U, and analyzed by EMSA. Inserts were derived from *FTZ* S/MAR, *Kcnd2* upstream intergenic region, Satellite I repeat (*Sat* I), and a randomly cloned *E*. *coli* DNA fragment (used as non-S/MAR control). To characterize the inserts, whose GC content, fragment length and C_50_ values are summarized at the bottom of the gel image. GC% for the vectors, pBS and pZErO, are 50.4 and 54.1, respectively. **(**B) Relative band densities for the vector and the insert bands were plotted against the concentration of hnRNP U. The dashed lines show the 50% level of unshifted band densities and arrow heads show the C_50_ of inserts. Maps of the recombinants used here is summarized in S2 Fig.
(PDF)

S2 Fig Map of plasmids used in S1 Fig. (A) pBS-FTZ (B) pBS-Kcnd2 intergenic region (C) pZErO-Sat I DNA (D) pBS-*E*. *coli* DNA are shown. Restriction enzyme sites to separate the vector and the insert are indicated with nucleotide number. The insert region and the selection marker in vectors are painted blue and yellow, respectively.
(PDF)

S3 Fig Analysis of rat hnRNP U 3D structure on AlphaFold program (v2.0 pipeline). The sequence used for analysis is rat hnRNP U in UniProt database (Q6IMY8). Shown here is a “predicted alignment error plot”. The shade of green indicates expected distance error in Ångströms. The color at (x, y) corresponds to the expected distance error in residue x’s position, when the prediction and true structure are aligned on residue y. Namely, dark green is good (low error) and light green is bad (high error). This diagram clearly shows that hnRNP U has folded structure in two positions. The small region close to N terminus coincides with SAP domain, whereas RG domain is located within the C-terminal disordered region. Presence of a long stretch of disordered segment between SAP domain and central structured domain may increase the mobility of SAP domain and thus facilitates the interaction with RG domain.
(PDF)

S4 Fig Purified recombinant proteins analyzed on SDS-PAGE. His-Myc-tagged or GST-fused proteins illustrated in Fig 2A were run on SDS-PAGE and stained with Coomassie Brilliant Blue. In each panel, gel images of proteins labeled on top are shown on the left, accompanied by uncropped images on the right. The cropped areas are boxed on the raw SDS-PAGE images. Amounts of proteins applied on to gels were 330, 770, 510, 300, 510, 360 and 100 ng for WT, RK, ΔRG, ΔSAP, NC, NCΔRG and N659, respectively. Proteins applied were 3.0 mg, 2.5 mg and 410 ng for GST-fused SAP, RG, and SAP-RG, respectively. M stands for molecular weight marker.
(PDF)

S5 Fig Uncropped agarose gel images used in this study. Cropped regions are boxed with black lines. For Fig 4A and 4B, ‘Left’ and ‘Right’ on top of the images indicate their positions in the figure.
(PDF)

S1 Table List of PCR primers used for plasmid construction. (XLSX)
