# Supplementary material for: Selective DNA-binding of SP120 (rat ortholog of human hnRNP U) is mediated by arginine-glycine rich domain and modulated by RNA
Source: PLoS One. 2023 Aug 4;18(8):e0289599. doi: 10.1371/journal.pone.0289599 (PMC10403129; doi:10.1371/journal.pone.0289599)
Supplement: S1 Fig — (A) Competitive EMSA analysis with equimolar mixtures of vectors and featured inserts. Twenty-five ng each of DNA were cleaved into vector (V) and insert (I) fragments, incubated with varying amounts of tag-purified hnRNP U, and analyzed by EMSA. Inserts were derived from FTZ S/MAR, Kcnd2 upstream intergenic region, Satellite I repeat (Sat I), and a randomly cloned E. coli DNA fragment (used as non-S/MAR control). To characterize the inserts, whose GC content, fragment length and C50 values are summarized at the bottom of the gel image. GC% for the vectors, pBS and pZErO, are 50.4 and 54.1, respectively. (B) Relative band densities for the vector and the insert bands were plotted against the concentration of hnRNP U. The dashed lines show the 50% level of unshifted band densities and arrow heads show the C50 of inserts. Maps of the recombinants used here is summarized in S2 Fig. (PDF) [file pone.0289599.s001.pdf]

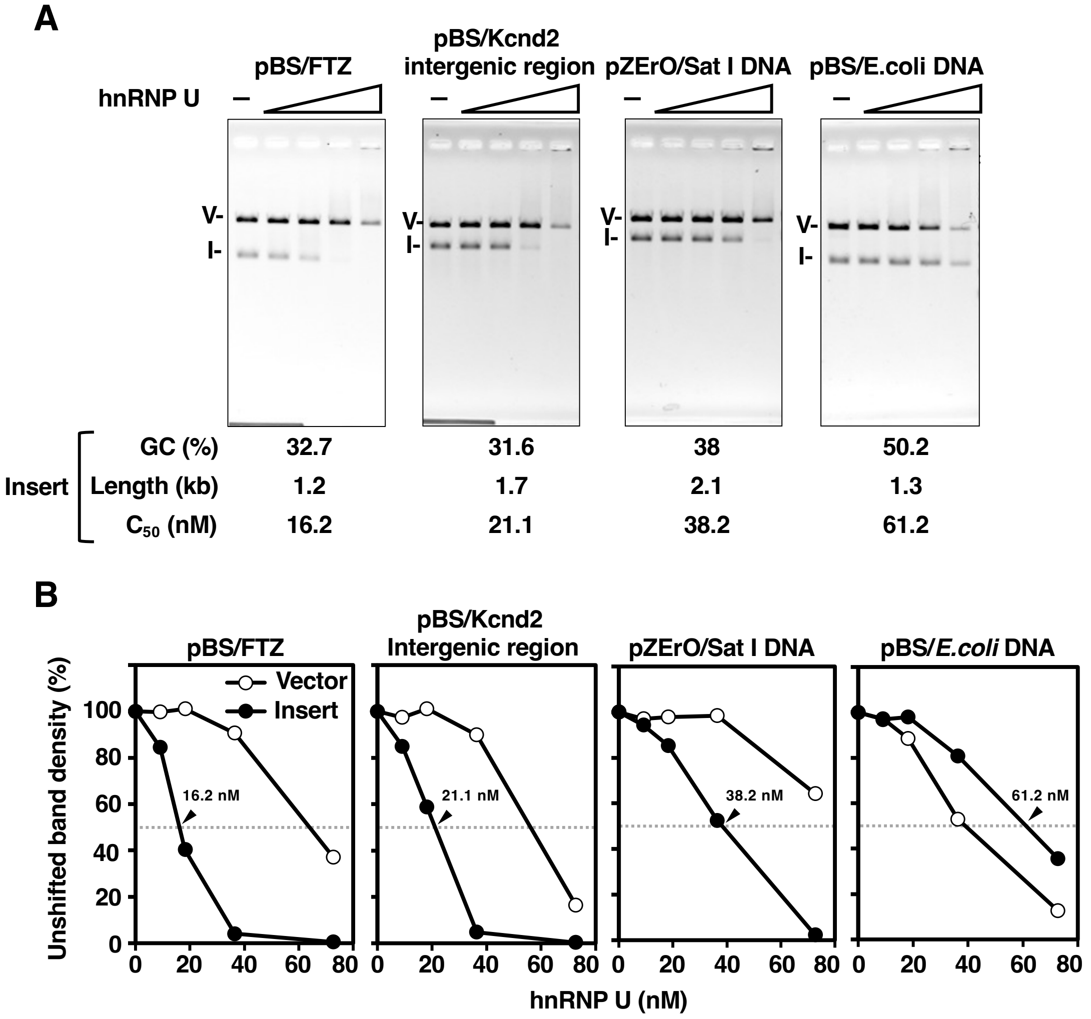

S1 Fig. S/MAR-selective binding activity of hnRNP U detected by magnesium-agarose EMSA. (A) Competitive EMSA analysis with equimolar mixtures of vectors and featured inserts. Twenty-five ng each of DNA were cleaved into vector (V) and insert (I) fragments, incubated with varying amounts of tag-purified hnRNP U, and analyzed by EMSA. Inserts were derived from *FTZ* S/MAR, *Kcnd2* upstream intergenic region, Satellite I repeat (*Sat* I), and a randomly cloned *E. coli* DNA fragment (used as non-S/MAR control). To characterize the inserts, whose GC content, fragment length and C_{50} values are summarized at the bottom of the gel image. GC% for the vectors, pBS and pZErO, are 50.4 and 54.1, respectively. (B) Relative band densities for the vector and the insert bands were plotted against the concentration of hnRNP U. The dashed lines show the 50% level of unshifted band densities and arrow heads show the C_{50} of inserts. Maps of the recombinants used here is summarized in S2 Fig.
