# Supplementary material for: Selective DNA-binding of SP120 (rat ortholog of human hnRNP U) is mediated by arginine-glycine rich domain and modulated by RNA
Source: PLoS One. 2023 Aug 4;18(8):e0289599. doi: 10.1371/journal.pone.0289599 (PMC10403129; doi:10.1371/journal.pone.0289599)
Supplement: S2 Fig — (A) pBS-FTZ (B) pBS-Kcnd2 intergenic region (C) pZErO-Sat I DNA (D) pBS-E. coli DNA are shown. Restriction enzyme sites to separate the vector and the insert are indicated with nucleotide number. The insert region and the selection marker in vectors are painted blue and yellow, respectively. (PDF) [file pone.0289599.s002.pdf]

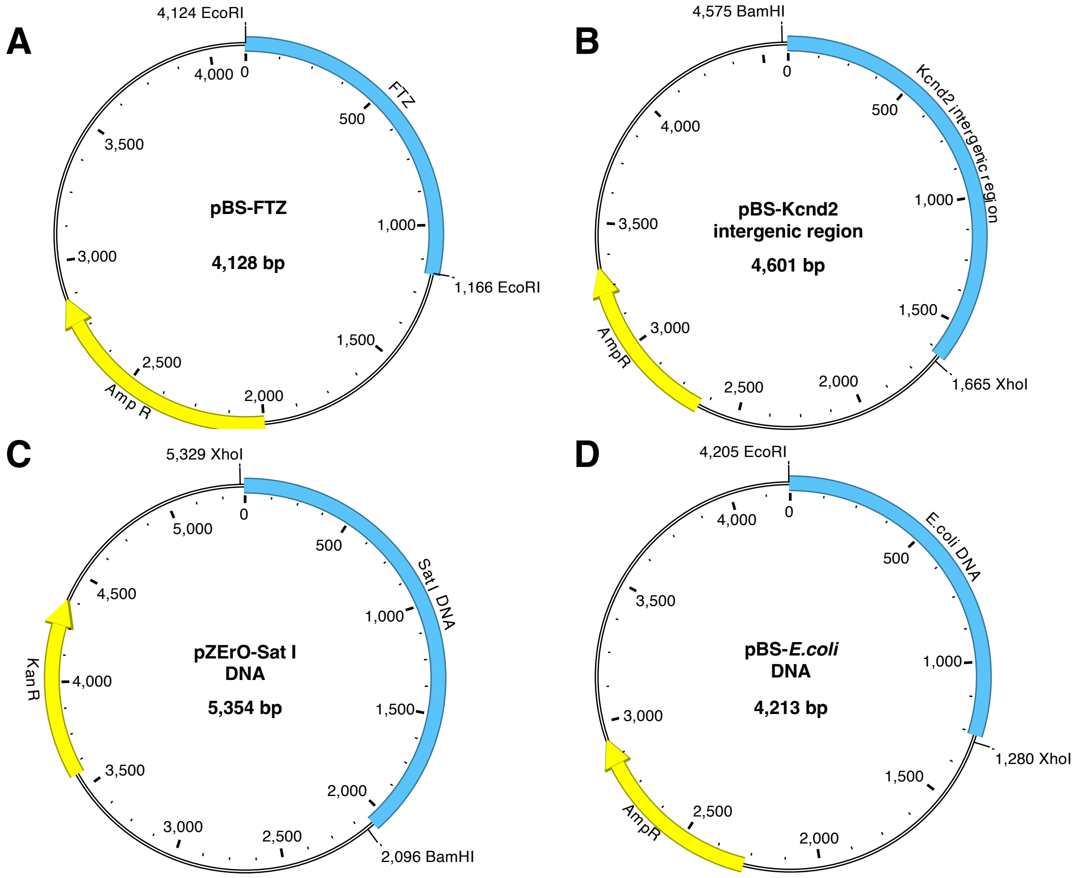

S2 Fig. Map of plasmids used in S1 Fig. (A) pBS-FTZ (B) pBS-Kcnd2 intergenic region (C) pZErO-Sat I DNA (D) pBS-*E. coli* DNA are shown. Restriction enzyme sites to separate the vector and the insert are indicated with nucleotide number. The insert region and the selection marker in vectors are painted blue and yellow, respectively.
