# Supplementary material for: Selective DNA-binding of SP120 (rat ortholog of human hnRNP U) is mediated by arginine-glycine rich domain and modulated by RNA
Source: PLoS One. 2023 Aug 4;18(8):e0289599. doi: 10.1371/journal.pone.0289599 (PMC10403129; doi:10.1371/journal.pone.0289599)
Supplement: S3 Fig — The sequence used for analysis is rat hnRNP U in UniProt database (Q6IMY8). Shown here is a “predicted alignment error plot”. The shade of green indicates expected distance error in Ångströms. The color at (x, y) corresponds to the expected distance error in residue x’s position, when the prediction and true structure are aligned on residue y. Namely, dark green is good (low error) and light green is bad (high error). This diagram clearly shows that hnRNP U has folded structure in two positions. The small region close to N terminus coincides with SAP domain, whereas RG domain is located within the C-terminal disordered region. Presence of a long stretch of disordered segment between SAP domain and central structured domain may increase the mobility of SAP domain and thus facilitates the interaction with RG domain. (PDF) [file pone.0289599.s003.pdf]

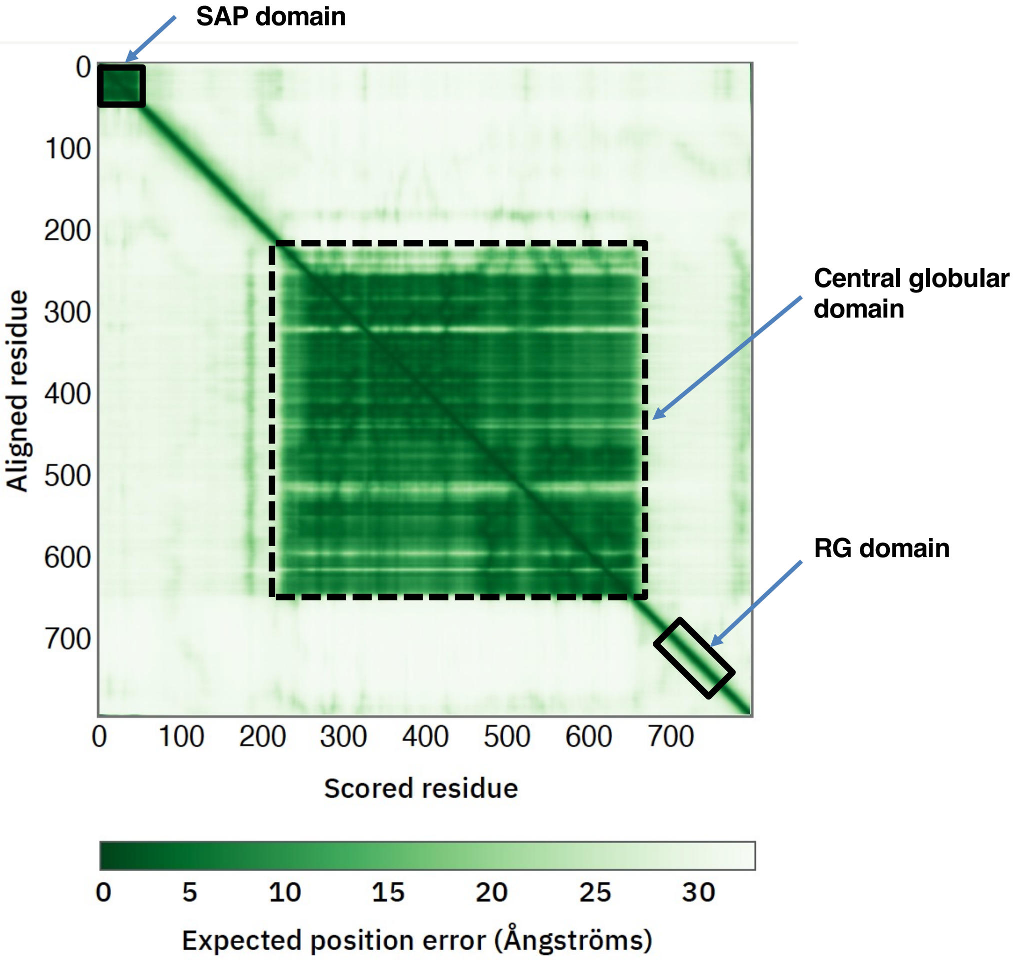

S3 Fig. Analysis of rat hnRNP U 3D structure on AlphaFold program (v2.0 pipeline). The sequence used for analysis is rat hnRNP U in UniProt database (Q6IMY8). Shown here is a "predicted alignment error plot". The shade of green indicates expected distance error in Ångströms. The color at (x, y) corresponds to the expected distance error in residue x's position, when the prediction and true structure are aligned on residue y. Namely, dark green is good (low error) and light green is bad (high error). This diagram clearly shows that hnRNP U has folded structure in two positions. The small region close to N terminus coincides with SAP domain, whereas RG domain is located within the C-terminal disordered region. Presence of a long stretch of disordered segment between SAP domain and central structured domain may increase the mobility of SAP domain and thus facilitates the interaction with RG domain.
