# Supplementary material for: Selective DNA-binding of SP120 (rat ortholog of human hnRNP U) is mediated by arginine-glycine rich domain and modulated by RNA
Source: PLoS One. 2023 Aug 4;18(8):e0289599. doi: 10.1371/journal.pone.0289599 (PMC10403129; doi:10.1371/journal.pone.0289599)
Supplement: S4 Fig — His-Myc-tagged or GST-fused proteins illustrated in Fig 2A were run on SDS-PAGE and stained with Coomassie Brilliant Blue. In each panel, gel images of proteins labeled on top are shown on the left, accompanied by uncropped images on the right. The cropped areas are boxed on the raw SDS-PAGE images. Amounts of proteins applied on to gels were 330, 770, 510, 300, 510, 360 and 100 ng for WT, RK, ΔRG, ΔSAP, NC, NCΔRG and N659, respectively. Proteins applied were 3.0 mg, 2.5 mg and 410 ng for GST-fused SAP, RG, and SAP-RG, respectively. M stands for molecular weight marker. (PDF) [file pone.0289599.s004.pdf]

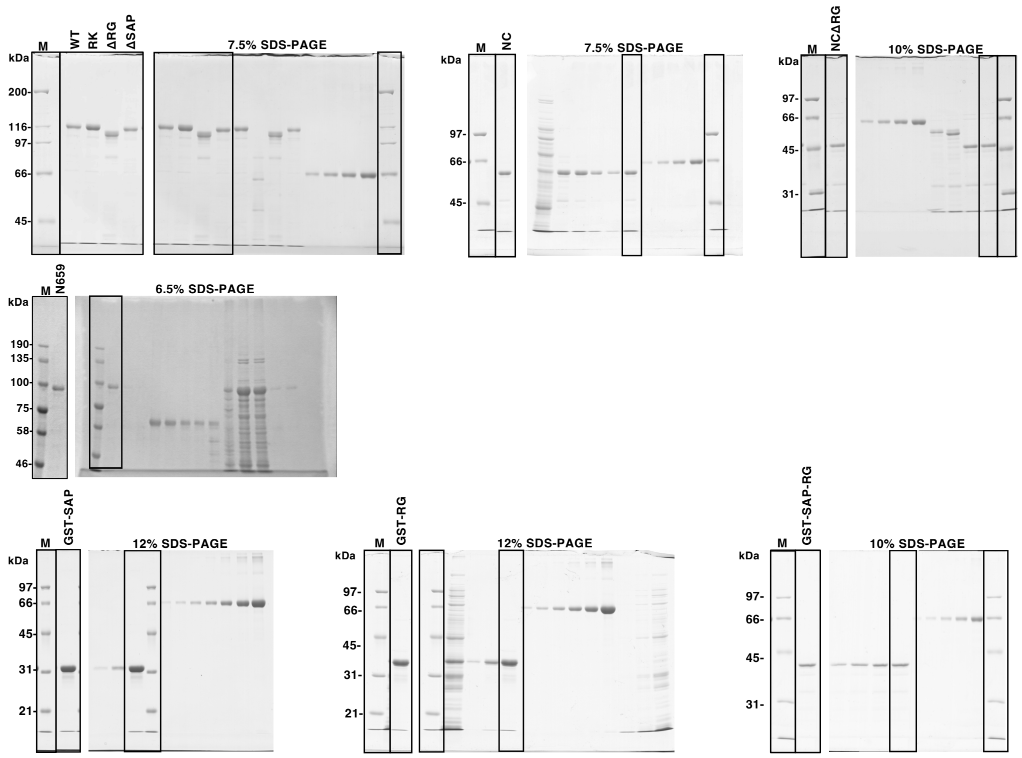

S4 Fig. Purified recombinant proteins analyzed on SDS-PAGE. His-Myc-tagged or GST-fused proteins illustrated in Fig 2A were run on SDS-PAGE and stained with Coomassie Brilliant Blue. In each panel, gel images of proteins labeled on top are shown on the left, accompanied by uncropped images on the right. The cropped areas are boxed on the raw SDS-PAGE images. Amounts of proteins applied on to gels were 330, 770, 510, 300, 510, 360 and 100 ng for WT, RK, ΔRG, ΔSAP, NC, NCΔRG and N659, respectively. Proteins applied were 3.0 μg, 2.5 μg and 410 ng for GST-fused SAP, RG, and SAP-RG, respectively. M stands for molecular weight marker.
