# Supplementary material for: Selective DNA-binding of SP120 (rat ortholog of human hnRNP U) is mediated by arginine-glycine rich domain and modulated by RNA
Source: PLoS One. 2023 Aug 4;18(8):e0289599. doi: 10.1371/journal.pone.0289599 (PMC10403129; doi:10.1371/journal.pone.0289599)
Supplement: S5 Fig — Cropped regions are boxed with black lines. For Fig 4A and 4B, ‘Left’ and ‘Right’ on top of the images indicate their positions in the figure. (PDF) [file pone.0289599.s005.pdf]

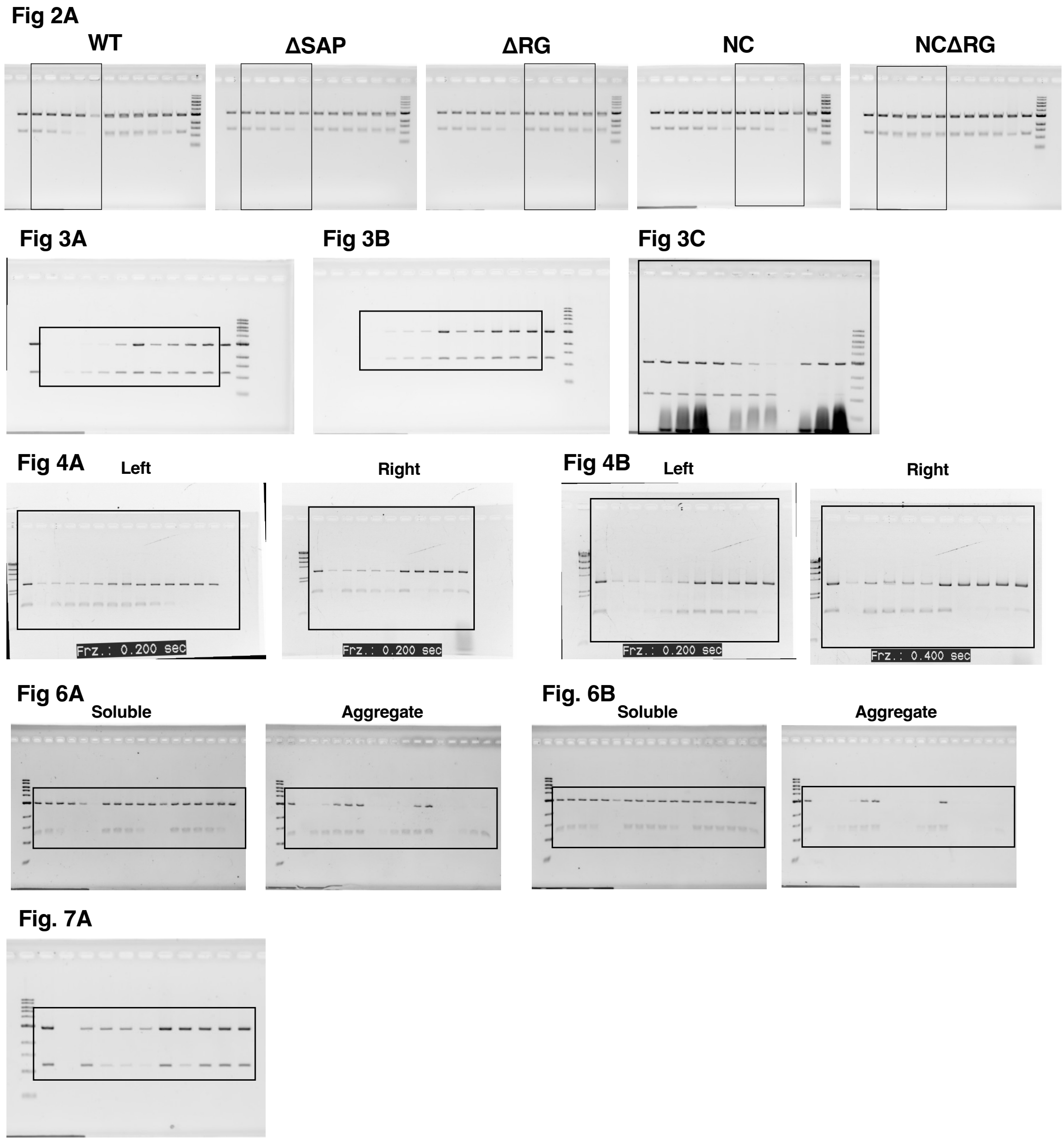

S5 Fig. Uncropped agarose gel images used in this study. Cropped regions are boxed with black lines. For Figs 4A and 4B, 'Left' and 'Right' on top of the images indicate their positions in the figure.
